# Molecular detection and assemblage analysis of the intestinal protozoan *Giardia duodenalis* in wild boars in Korea

**DOI:** 10.3389/fvets.2023.1139060

**Published:** 2023-04-18

**Authors:** Haeseung Lee, Dongmi Kwak

**Affiliations:** College of Veterinary Medicine, Kyungpook National University, Daegu, Republic of Korea

**Keywords:** genotype, infection rate, phylogenetic analysis, wild boar, *Giardia duodenalis*

## Abstract

*Giardia duodenalis* (syn. *G. intestinalis, G. lamblia*) is the only *Giardia* species that infects humans and most other mammals. Wild boars are a reservoir of many viruses, bacteria, and parasites that can be transmitted to livestock and humans. This study examined the infection rate of *G. duodenalis* in wild boars and confirmed its specificity by comparing assemblages through PCR amplification of the 18S rRNA, *gdh*, and β-giardin genes. Fecal samples were collected from roadkilled or trapped wild boars from April 2016 to December 2021 in Korea. DNA was extracted directly from 612 wild boar fecal specimens using a commercial kit. PCR was performed targeting the 18S rRNA region, β-giardin, and glutamate dehydrogenase genes of *G. duodenalis.* Some PCR-positive samples were selected for sequencing analysis. The obtained sequences were subsequently used for phylogenetic tree construction. Of the 612 samples tested, 125 (20.4%) were positive for *G. duodenalis.* The highest infection rate was detected in the central region (12.0%) and in autumn (12.7%). Among the risk factors, the seasonal factor was statistically significant (*p* = 0.012). Phylogenetic analysis revealed three genetic assemblages: A, B, and E. Assemblages A and B exhibited 100% identity with *Giardia* sequences isolated from human and farmed pigs in Korea and Japan. This result cannot be ignored because it indicates the possibility of zoonotic transmission. Therefore, continuous management and monitoring of this pathogen are necessary to prevent transmission and protect animal and human health.

## 1. Introduction

*Giardia* is a flagellated protozoan parasite that infects various vertebrates (1, 2). Currently, seven nonhuman-infecting species (*Giardia agilis*, *G. ardaea*, *G. psittaci*, *G*. *muris*, *G*. *microti*, *G*. *peramelis,* and *G*. *cricetidarum*) and one species infecting humans and other mammals, *Giardia duodenalis* (syn. *G*. *intestinalis*, *G*. *lamblia*), have been identified (2, 3).

The infection caused by *G*. *duodenalis*—known as giardiasis—is important from veterinary and public health perspectives. This parasite has a wide range of hosts, including wild animals, and giardiasis is a common disease in livestock and companion animals (1, 4, 5). Additionally, *Giardia* infections are prevalent in areas with poor hygiene, where the ingestion of cysts is high. However, cases are emerging worldwide because infection occurs when cysts are ingested through contaminated water or direct person-to-person contact (6–10).

Molecular studies have classified *G. duodenalis* into eight distinct genetic groups, known as assemblages A–H (3, 5, 7–9). These assemblages are morphologically similar but exhibit genetic heterogeneity (7). Assemblages A and B are predominant in humans; however, they have been reported to have zoonotic potential as they have been detected in several other mammals, and their host range is comprehensive (3, 4, 7, 11, 12). In contrast, assemblages C–H have been identified in nonhuman hosts (4, 6, 11, 12), with a few exceptions (13–15). Assemblages C and D have been identified in canines, E in hoofed livestock, F in cats, G in rodents, and H in pinnipeds (12, 15, 16).

Wild boars are widely distributed worldwide and are edible wild animals. However, they are susceptible to several parasites (e.g., helminths/protozoa, viruses, or bacteria), making them potential reservoirs for disease transmission (17, 18). Some studies have investigated the relationship between wild boar contact and disease transmission and the role played by wild boars in foodborne zoonoses (19, 20). Studies of *Giardia* infections in wild boars and domestic pigs have been conducted worldwide (17, 21–24). However, research on *Giardia* in Korea has mainly focused on environmental samples—including drinking water, cattle-like livestock animals, and companion animals, such as dogs—and studies on wild animals are lacking (25–29). No studies have been conducted on *Giardia* infections in wild boars, and only one study is available on domestic pigs (30).

Therefore, this study aimed to confirm the rate of *Giardia* infection, genetic diversity, and potential for zoonotic transmission in wild boars and to compare them with *Giardia* assemblages in domestic pigs.

## 2. Materials and methods

### 2.1. Study area and collection of fecal samples

**Figure 1:**
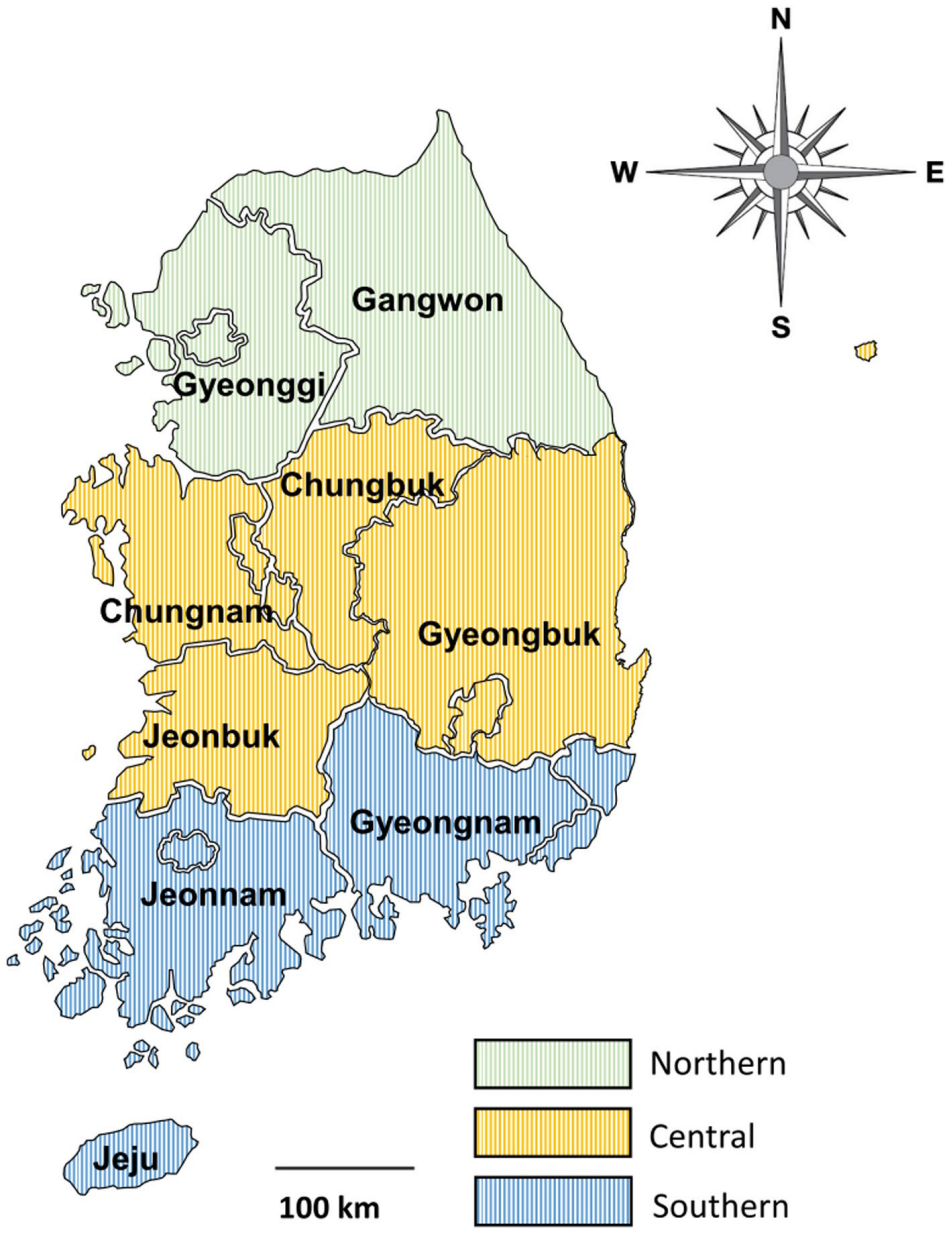
Map of the sample collection regions. The country was divided into three regions based on the boundaries of administrative districts as follows: northern (provinces of Gangwon and Gyeonggi), central (provinces of Chungbuk, Chungnam, Gyeongbuk, and Jeonbuk), and southern (provinces of Gyeongnam, Jeju, and Jeonnam).

From April 2016 to December 2021, fecal samples were collected from deceased wild boars found trapped in forests or on roads after being hit by vehicles across the country. Wild boar feces were collected from the intestines after a veterinary performed the carcass necropsy. This procedure was supervised by the National Institute of Environmental Research in Korea. Because the collection of feces from the carcass was unrelated to research ethics and did not cause hazards to any animals, approval from Kyungpook National University’s Institutional Animal Care and Use Committee was not required for the present study. Samples were individually placed in tubes and delivered to the laboratory, where DNA was extracted. The primary data for fecal samples, including region, season, and sex, were recorded for each individual, and any unclear or suspicious data were logged as “unknown.” The samples were collected in spring, summer, and autumn. No samples were collected in winter. The sampled areas were divided into three regions—northern, central, and southern—based on the boundaries of the administrative districts (Figure 1).

### 2.2. DNA extraction and PCR assay

DNA was extracted using a commercially available kit (QIAamp® Fast DNA Stool Mini Kit; QIAGEN, Hilden, Germany) according to the manufacturer’s protocol. The extracted DNA was placed in a sterile tube and stored at −20°C.

Nested PCR was performed to amplify the three target genes related to *Giardia* detection. Initially, primers amplifying the 18S rRNA region were used to screen positive samples. The primers used were RH11 (5′-CAT CCG GTC GAT CCT GCC-3′) and RH4LM (5′-GTC GAA CCC TGA TTC TCC G-3′) in the first round and GiarF (5′-GAC GCT CTC CCC AAG GAC-3′) and GiarR (5′-CTG CGT CAC GCT GCT CG-3′) in the second round (31). These primer sets were used to amplify the 18S rRNA gene for a length of approximately 170 bp. A commercial premix kit (AccuPower® HotStart PCR PreMix; Bioneer, Daejon, Korea) was used for regular PCR. The reaction was conducted in a volume of 20 μL containing 1 μL of each primer, 3 μL of sample DNA or first-round PCR products, and 15 μL of sterile distilled water. PCR amplifications were performed using Mastercycler nexus (Eppendorf, Hamburg, Germany) with the following conditions: initial denaturation at 95°C for 5 min; 35 cycles of denaturation, annealing, and extension at 95°C for 30 s, 59°C for 30 s, and 72°C for 20 s, respectively; and final extension at 72°C for 5 min.

Subsequently, additional PCR was performed to obtain positive sequences by amplifying the β-giardin (*bg*), glutamate dehydrogenase (*gdh*), and triosephosphate isomerase (*tpi*) genes of the *Giardia*-positive DNA samples screened *via* 18S rRNA PCR. Nested PCR was performed to amplify three genes. The expected fragment sizes were approximately 510 bp for *bg* (32, 33) and approximately 530 bp for *gdh* and *tpi* (34). For the PCR amplification of *gdh*, gdh1/2 (TTC CGT RTY CAG TAC AAC TC/ACC TCG TTC TGR GTG GCG CA) and gdh3/4 (ATG ACY GAG CTY CAG AGG CAC GT/GTG GCG CAR GGC ATG ATG CA) primer sets were used. The PCR conditions were as follows: initial denaturation at 95°C for 5 min, followed by 35 cycles of denaturation, annealing, and extension at 95°C for 30 s, 55°C for 30 s (nested PCR at 59°C for 30 s), and 72°C for 30 s, respectively, and a final extension at 72°C for 5 min. For the PCR amplification of *bg*, G7/G759 (AAG CCC GAC GAC CTC ACC CGC AGT GC/GAG GCC GCC CTG GAT CTT CGA GAC GAC) and G7n/G759n (GAA CGA GAT CGA GGT CCG/CTC GAC GAG CTT CGT GTT) primer sets were used. The PCR conditions were as follows: initial denaturation at 95°C for 5 min, followed by 35 cycles of denaturation, annealing, and extension at 95°C for 30 s, 65°C for 30 s (nested PCR at 55°C for 30 s), and 72°C for 30 s, respectively, and a final extension at 72°C for 5 min. For the PCR amplification of *tpi*, gtp1/2 (AAA TIA TGC CTG CTC GTC G/CAA ACC TTI TCC GCA AAC C) and gtp3/4 (CCC TTC ATC GGI GGT AAC TT/GTG GCC ACC ACI CCC GTG CC) primer sets were used. The reaction conditions included an initial denaturation at 95°C for 5 min; 35 cycles of denaturation, annealing, and extension at 95°C for 30 s, 50°C for 30 s, and 72°C for 30 s, respectively, and a final extension at 72°C for 5 min. All PCR products were loaded in the electrophoresis unit with 1.5% agarose gel stained with ethidium bromide. The gel was run for 30 min at 135 V. Images were acquired using an ultraviolet transilluminator. PCR-positive samples were sent to Macrogen (Daejeon, Korea) for direct DNA sequencing.

### 2.3. Statistical and phylogenetic analysis

The data were subjected to χ^2^ test using SPSS version 26.0 (IBM Corporation, Armonk, NY, United States), and *p* values of <0.05 were considered statistically significant. Unknown data were excluded from the calculations as missing values.

For the phylogenetic analysis, MEGA7 software was used to construct each phylogenetic tree using the *Giardia* 18S rRNA, *bg*, and *gdh* sequences obtained in this study and the GenBank-accessed sequence. Phylogenetic inference was conducted using the maximum likelihood method with 1,000 bootstrap replications.

## 3. Results

### 3.1. *Giardia* infection rates in wild boars based on 18S rRNA amplification

The PCR results were evaluated based on region, season, and sex. An overall *Giardia* infection rate of 20.4% (125/612) was observed (Table 1).

**Table 1:** *Giardia duodenalis* infection in wild boars based on 18S rRNA amplification.

Group			No. tested	No. positive (%)	value of *p*
Sex	Male		227	21 (9.3)	0.544
	Female		166	15 (9.0)	
	Unknown		219	89 (40.6)	
Region^a^	Northern	GW	109	9 (8.3)	0.635
		GG	101	10 (9.9)	
		Subtotal	210	19 (9.0)	
	Central	CB	49	5 (10.2)	
		CN	32	3 (9.4)	
		GB	69	8 (11.6)	
		JB	33	6 (18.2)	
		Subtotal	183	22 (12.0)	
	Southern	GN	58	6 (10.3)	
		JN	32	3 (9.4)	
		JJ	4	0 (0.0)	
		Subtotal	94	9 (9.6)	
	Unknown		125	75 (60.0)	
Season	Spring		44	0 (0.0)	0.012
	Summer		202	19 (9.4)	
	Autumn		252	32 (12.7)	
	Unknown		111	73 (65.8)	
Total			612	125 (20.4)	

a CB, Chungbuk; CN, Chungnam; GB, Gyeongbuk; GG, Gyeonggi; GN, Gyeongnam; GW, Gangwon; JB, Jeonbuk; JJ, Jeju; and JN, Jeonnam.

The infection rates in the northern, central, and southern regions were 9.0% (19/210), 12.0% (22/183), and 9.6% (9/94), respectively, while the rate was 60.0% (72/125) for the local unknown sample. The infection rate in the central region was higher than that in other regions. However, the values did not differ significantly among groups, calculated by excluding unknown regional samples (*p* = 0.635). The infection rate was 9.4% (19/202) in summer and 12.7% (32/252) in autumn. No positivity was detected in spring. Among the 125 positive samples, 19 were identified in summer (15.2%) and 32 in autumn (25.6%). The differences in infection rates among the groups were statistically significant (*p* = 0.012). Infections were detected in 9.3% of males, 9.0% of females, and 40.6% of wild boars with unknown sex, with no significant differences among groups (*p* = 0.544).

### 3.2. Sequencing and phylogenetic analysis

**Figure 2:**
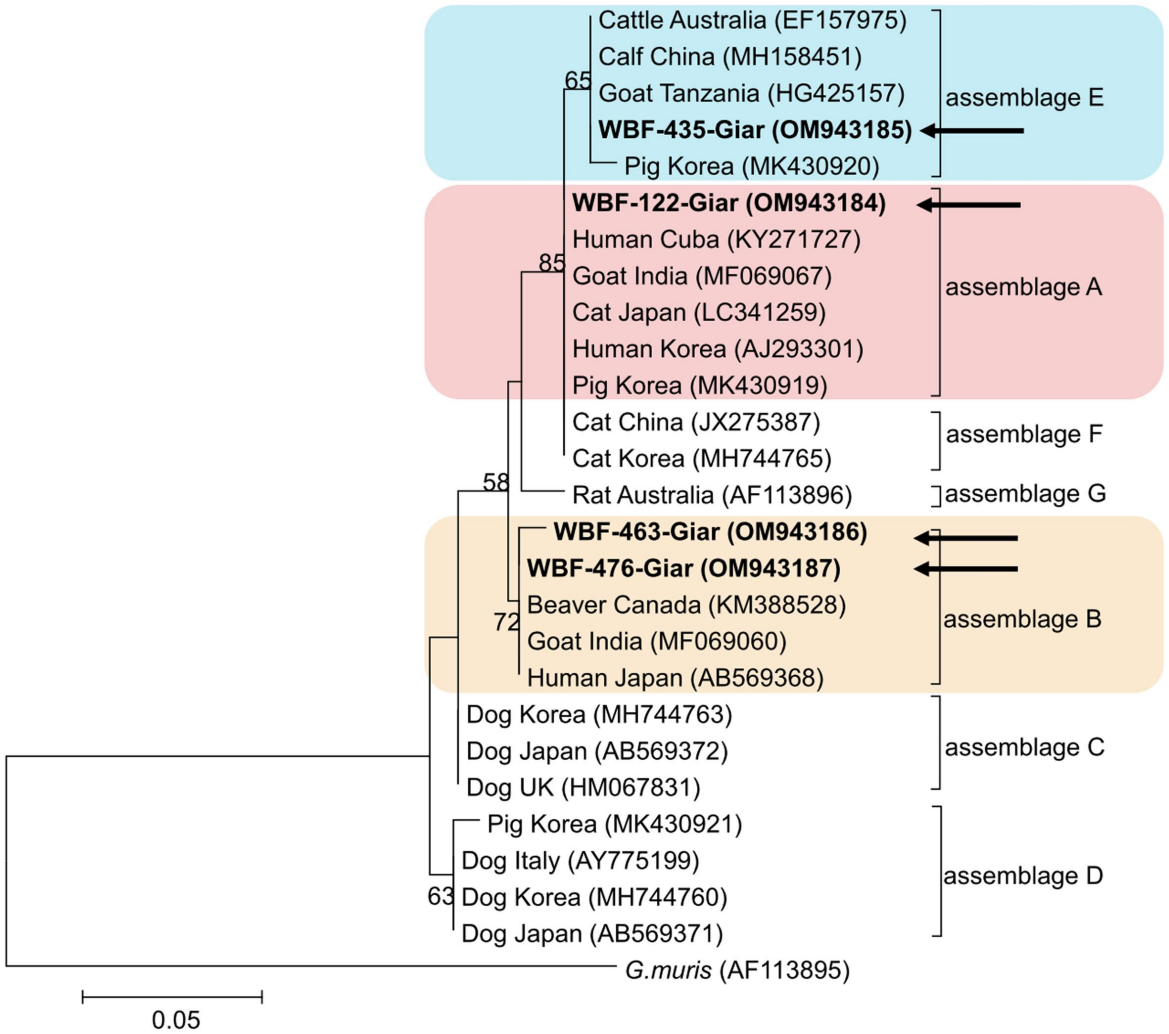
Phylogenetic tree of *Giardia* (18S rRNA) isolated from Korean wild boars. The sequences obtained from the phylogenetic analysis based on the *Giardia* 18S ribosomal RNA are indicated in bold.

**Figure 3:**
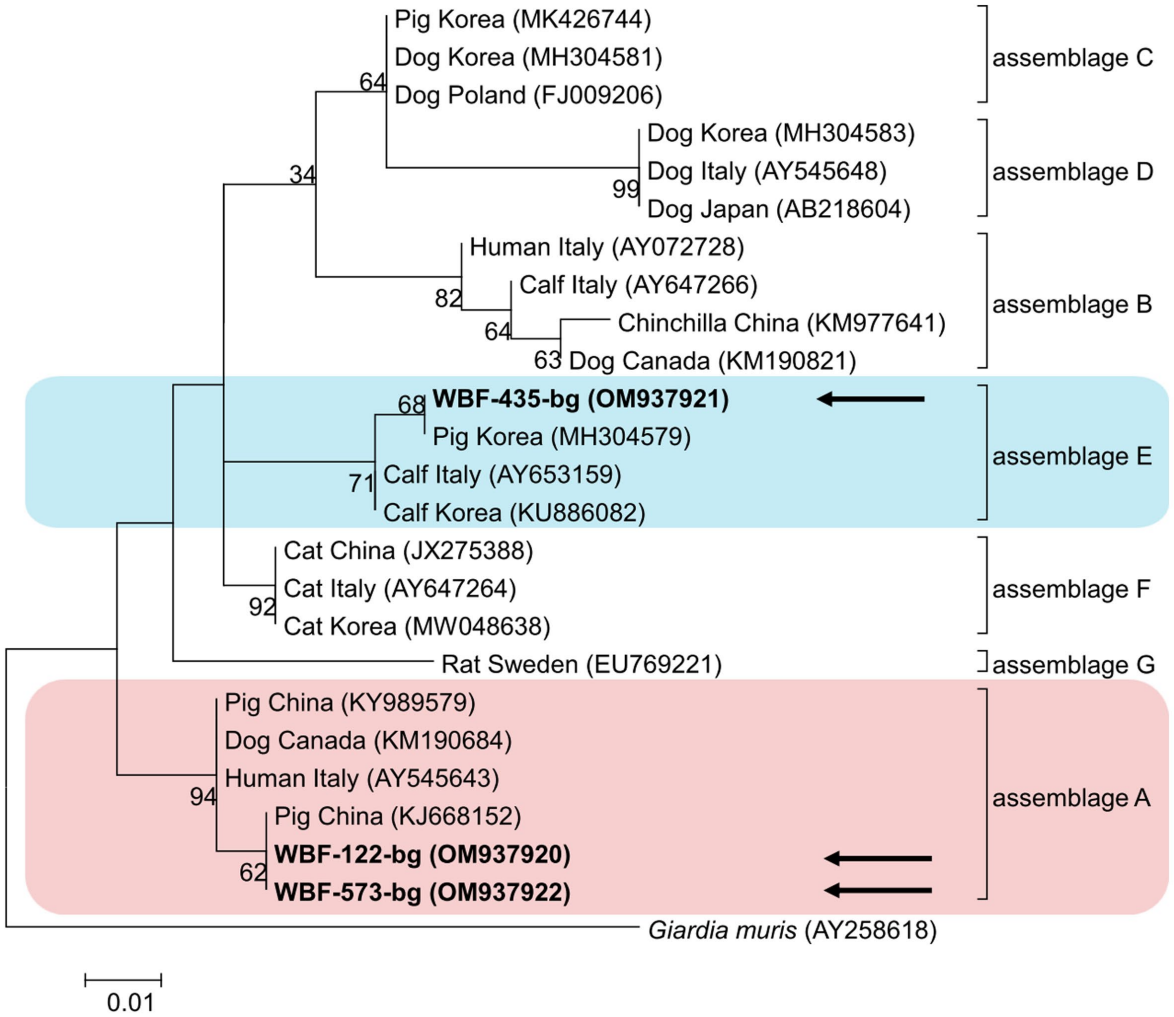
Phylogenetic tree of *Giardia* (β-giardin) isolated from Korean wild boars. The sequences obtained from the phylogenetic analysis based on the *Giardia* β-giardin gene are indicated in bold.

**Figure 4:**
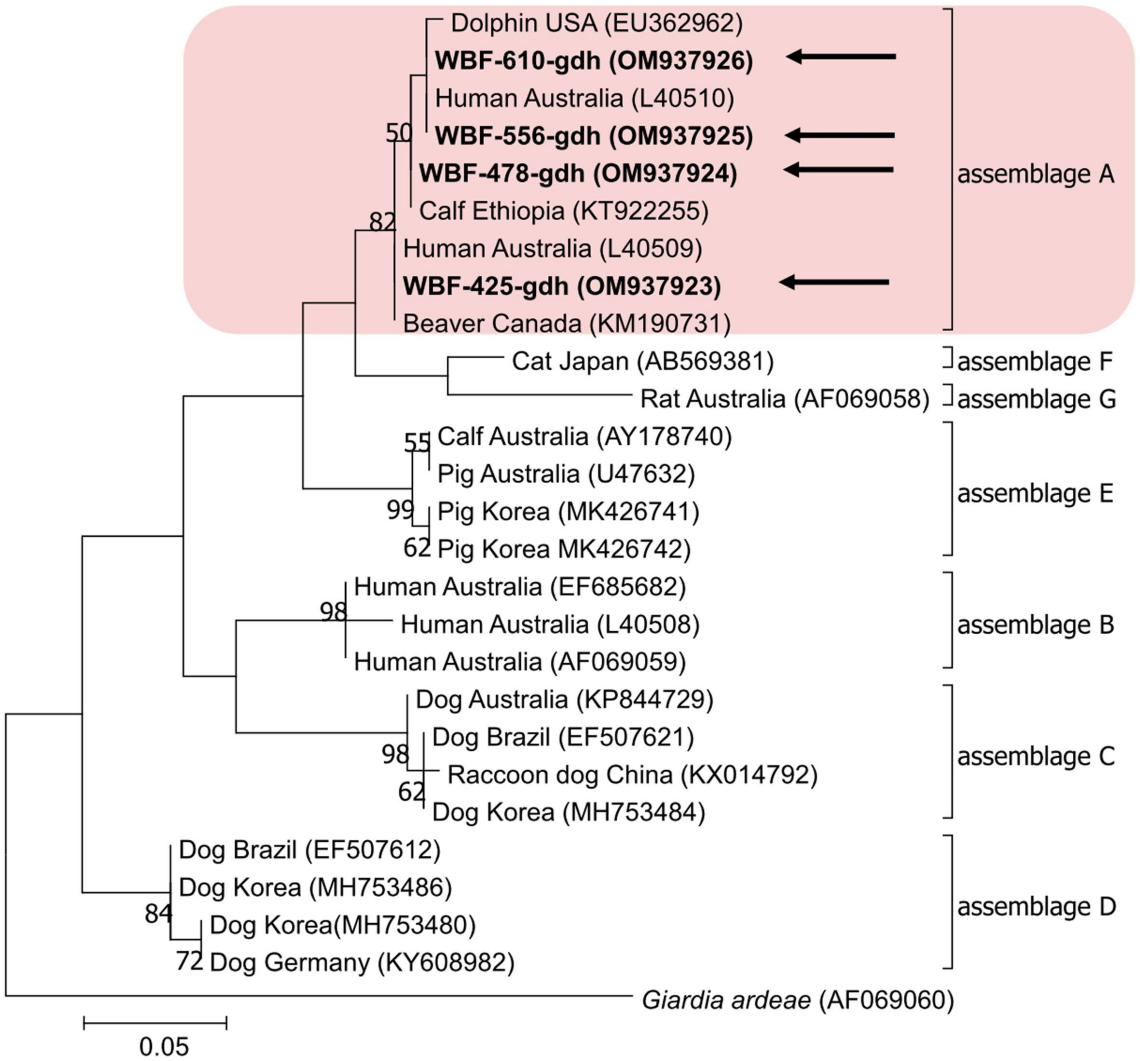
Phylogenetic tree of *Giardia* (gdh) isolated from Korean wild boars. The sequences obtained from the phylogenetic analysis based on the *Giardia* gdh gene are indicated in bold.

Of the 125 18S rRNA-positive samples, we obtained 19 successfully aligned nucleotide sequences through 18S rRNA sequencing. Phylogenetic analysis based on the *Giardia* 18S rRNA sequence revealed three assemblages: A, B, and E. Four sequences (OM943184–OM943187) from positive samples and a reference sequence obtained from GenBank were used for phylogenetic analysis. The obtained sequences are shown in bold in Figure 2. Three *bg* locus-positive (2.4%, 3/125) samples (OM937920–OM937922) and four *gdh* locus-positive (3.2%, 4/125) samples (OM937923–OM937926) were successfully sequenced, and assemblages A and E were confirmed following phylogenetic analyses based on the sequences of *bg* and *gdh* (Table 2; Figures 3, 4). However, *tpi* was not detected in any of the samples.

**Table 2:** Genotyping of *Giardia duodenalis* based on 18S rRNA, *β-giardin*, and *gdh* amplification.

Genotypes	*ssu* rRNA (*n* = 19)	*β-giardin* (*n* = 3)	*gdh* (*n* = 4)
Assemblage A	16	2	4
Assemblage B	2	0	0
Assemblage E	1	1	0

## 4. Discussion

*Giardia* infection rates have been studied in many countries, both in humans and animals (5, 7, 11, 15, 35). Notably, the rates of *Giardia* infection in wild boars have been reported to be 40.7% in Poland, 22.5% in southern Spain, 3.1% in China, 1.7% in Croatia, and 1.3% in northwestern Spain (17, 23, 24, 36, 37). However, no studies have been conducted on *Giardia* infection in Korean wild boars. Even when extending the scope of investigation to livestock pigs, only one infection study has been conducted, with a reported infection rate of 14.8% (110/745) (30). However, this result was obtained from a survey targeting only the central and southern regions of Korea. The infection rate in wild boars confirmed in the present study was 20.4% (125/612), which is higher than that in other animals (e.g., livestock and companion animals) in Korea. For instance, infection rates in calves were reported to be 13.1% (77/590) in 2016 (29), 12.7% (40/315) in 2018 (38), and 5.6% (44/792) in 2021 (39). The value was 3.8% in cats (6/158) (40) and 15.5% (99/640) in dogs (26). Only one study of Korean wild animals was conducted with small sample sizes, and the following infection rates were reported: 14.3% (1/7) in Eurasian otter, 31.8% (7/22) in leopard cat, and 9.1% (1/11) in raccoon dog (27). Various factors, such as the host, number of samples, country or region, surrounding environment, and diagnostic methods, result in differences in the obtained values. For example, a large difference in the positivity rate between direct fluorescence assay and PCR methods has been reported in a study conducted in Poland (14.8% vs. 40.7%, respectively) (37).

In the present study, DNA sequences were extracted from 612 fecal samples and analyzed based on the sampling region, season, and sex using PCR. The infection rates were the highest in the central region (12.0%) and the lowest in the northern region (9.0%). However, this difference was not statistically significant, which is in contrast with the results of previous studies conducted on dogs and pigs (26, 30). In pigs, the infection rate in the southern region (16.3%) was reported to be twice that in the central region (8.1%), which was statistically significant. In dogs, the infection rate in the southern region (40.7%) was significantly higher than that in other regions (northern 11.5%, central 7.9%). Therefore, it is assumed that other external factors within the entire area, such as the living environment of the host, may cause differences in the infection rate by region, and additional research is needed to explore this hypothesis further.

With regard to season, the results revealed a positive rate of 0.0% in spring, 9.4% in summer, and 12.7% in autumn. However, the lack of winter samples and the absence of infections in spring require further validation through additional samples. *Giardia* infection rates have been reported to be the highest during the rainy season in several previous studies (41, 42), confirming that the illness is waterborne. Furthermore, Lee et al. (30) reported higher infection rates in autumn (21.2%) than in summer (12.0%) for pigs, which is consistent with the results of wild boar infections reported in our study.

In terms of sex, the infection rates did not differ significantly between male (9.3%) and female wild boars (9.0%), which is consistent with the results reported for Nigerian pigs (21), where the infection rate was 25.0% for males and 25.7% for females. The infection rates did not differ significantly in studies conducted on other animals in Korea. The values were 17.6 and 13.8% for male and female dogs, respectively, and 4.8 and 3.1% for male and female cats, respectively (26, 40). However, the relationship between *Giardia* infection and sex remains unclear, as most infections were detected in wild boars of unknown sex (40.6%). Therefore, further studies are needed to clarify this aspect.

In this study, *bg* or *gdh* showed a lower positivity rate than 18S rRNA; even *tpi* was not detected. This is a characteristic of the protein-coding gene. Because the protein-coding gene is a single-copy gene (43), previous studies of *Giardia* infection in Korean dogs and pigs showed low sensitivity (26, 30). Nevertheless, it was used to compare the genotype results of 18S rRNA and *bg* or *gdh* with each other.

In the present study, the genotypes obtained based on 18S rRNA analysis were identified as assemblages A, B, and E. Assemblages A and B were distinguished as potential zoonotic groups, owing to their wide and diverse host ranges (12, 44). Assemblage A in wild boars was first identified in Croatia (23), whereas assemblage B was first identified in Poland (37). As few studies are available on wild boars, the results of studies on pigs were used for comparisons. Assemblage E was predominantly identified, assemblage A was partly identified, and assemblage B was rarely identified (5, 45–48). The results of a study on Korean pigs are similar (30). Assemblages A and E as well as C and D were confirmed in Korean pigs. Assemblage E was identified as the predominant one, while assemblage B was not detected (30). However, assemblage B, which had not been previously identified in pigs, was detected, and assemblage A was more common than the other assemblages. As the human infection of assemblage E has been confirmed in a recent Brazilian study (49), the possibility of zoonotic transmission in all types identified in this study should be considered. Notably, the same assemblage A was confirmed in the *bg* sequence (OM937920) in the case of sample WBF-122, and the WBF-122 18S rRNA sequence (OM943184), the Korean human sequence (AJ293301), and the Korean pig sequence (MK430919) showed 100% sequence similarity. Additionally, the WBF-122 *bg* (OM937920) and the Chinese pig (KJ668152) sequences showed 100 and 99.3% similarity to the Italian human sequence (AY545643), respectively. Assemblage A was also confirmed in the *gdh* sequence. The WBF-556 *gdh* sequence (OM937925) showed 99.8% similarity to the Australian sequence (L40510). Assemblage E was also identified in the sequences of 18S rRNA (OM943185) and *bg* (OM937921) of the WBF-435 sample. The WBF-435 *bg* sequence (OM937921) showed 100% identity with the sequence identified in Korean pigs (MH304579). However, the *gdh* sequences of other assemblages were not identified, possibly because the *bg* and *gdh* protein-coding genes have lower sensitivity than 18S rRNA (43). Therefore, this is a limitation of the study and a subject for future research.

## 5. Conclusion

The overall *Giardia* infection rate in Korean wild boars was 20.4%. This study analyzed differences in infection rates based on region, season, and sex to determine the risk factors for infection. Only season was identified as a statistically significant factor. However, assemblages A, B, and E were identified in the fecal samples, and assemblage A was confirmed to be 100% identical to the genotype found in human and farmed pigs in Korea. This indicates the possibility of *Giardia* transmission from a range of animals to other animals, or from animals to humans. Assemblages A and B were confirmed to be zoonotic, and assemblage E was confirmed to be zoonotic as well; however, human infections are rare. Therefore, their zoonotic potential should be studied.

To the best of our knowledge, this was the first nationwide study of *Giardia* infections affecting wild boars that provided basic data on genetic diversity. However, infection rates should be further confirmed by analyzing more samples. Additionally, comparative analysis with protein-coding genes should be conducted to identify genetic characteristics in future studies. The results obtained in this study, which indicate the possibility of zoonotic transmission, cannot be ignored. Furthermore, continuous management and monitoring of this pathogen are necessary to prevent transmission and protect the health of animals and humans.

## Data availability statement

The datasets presented in this study can be found in online repositories. The names of the repository/repositories and accession number(s) can be found at: https://www.ncbi.nlm.nih.gov/genbank/, OM943185; https://www.ncbi.nlm.nih.gov/genbank/, OM943184; https://www.ncbi.nlm.nih.gov/genbank/, OM943186; https://www.ncbi.nlm.nih.gov/genbank/, OM943187; https://www.ncbi.nlm.nih.gov/genbank/, OM937921; https://www.ncbi.nlm.nih.gov/genbank/, OM937920; https://www.ncbi.nlm.nih.gov/genbank/, OM937922; https://www.ncbi.nlm.nih.gov/genbank/, OM937926; https://www.ncbi.nlm.nih.gov/genbank/, OM937925; https://www.ncbi.nlm.nih.gov/genbank/, OM937924; and https://www.ncbi.nlm.nih.gov/genbank/, OM937923.

## Ethics statement

Since the collection of feces from carcass was not related to research ethics and did not cause hazard to any animals, the approval from Kyungpook National University’s Institutional Animal Care and Use Committee was not required for the present study.

## Author contributions

DK designed the study. HL conducted the experiment, wrote the manuscript, performed statistics, and analyzed the data. HL and DK edited the article. All authors contributed to the article and approved the submitted version.

## Conflict of interest

The authors declare that the research was conducted in the absence of any commercial or financial relationships that could be construed as a potential conflict of interest.

## Publisher’s note

All claims expressed in this article are solely those of the authors and do not necessarily represent those of their affiliated organizations, or those of the publisher, the editors and the reviewers. Any product that may be evaluated in this article, or claim that may be made by its manufacturer, is not guaranteed or endorsed by the publisher.
